# Tumor-Draining Lymph Node Reconstruction Promotes B Cell Activation During E0771 Mouse Breast Cancer Growth

**DOI:** 10.3389/fphar.2022.825287

**Published:** 2022-03-28

**Authors:** Dante Alexander Patrick Louie, Darellynn Oo, Glory Leung, Yujia Lin, Matthew Stephens, Omar Alrashed, Marcus Tso, Shan Liao

**Affiliations:** ^1^ Department of Microbiology, Immunology and Infectious Diseases, the Snyder Institute for Chronic Diseases, Cumming School of Medicine, University of Calgary, Calgary, AB, Canada; ^2^ Department of General Surgery, The Second Affiliated Hospital of Harbin Medical University, Harbin Medical University, Harbin, China

**Keywords:** Tumor-draining lymph node, B cell, breast cancer, subcapular sinus macrophage, germinal center (GC), tumor-associated antigen (TAA), follicular dendritic cell (FDC), Lymph node conduits

## Abstract

Lymph node metastasis is associated with tumor aggressiveness and poor prognosis in patients. Despite its significance in cancer progression, how immune cells in the tumor-draining lymph node (TDLN) participate in cancer immune regulation remains poorly understood. It has been reported that both anti-tumor and exhausted tumor-specific T cells can be induced in the TDLNs; however, B cell activation and maturation in the TDLN has received far less attention. In our studies using C57BL/6 mouse syngeneic E0771 breast cancer or B16F10 melanoma cell lines, tumor-associated antigens were found colocalized with the follicular dendritic cells (FDCs) in the germinal centers (GCs), where antigen-specific B cell maturation occurs. LN conduits and the subcapsular sinus (SCS) macrophages are two major routes of antigen trafficking to FDCs. Tumor growth induced LN conduit expansion in the B cell zone and disrupted the SCS macrophage layer, facilitating both the entry of tumor-associated antigens into the B cell zone and access to FDCs located in the GCs. Regional delivery of clodronate liposome specifically depleted SCS macrophages in the TDLN, increasing GC formation, and promoting tumor growth. Our study suggests that TDLN reconstruction creates a niche that favors B cell activation and maturation during tumor growth.

## Introduction

Lymph node (LN) metastasis is the most important criterion for patient diagnosis, prognosis, and treatment (Karpanen et al., 2006). In the tumor-draining LN (TDLN), metastatic cancer cells that escape the host’s immune surveillance can invade the blood vessels in the TDLN and spread to distant organs, demonstrating the importance of LN metastases in cancer progression (Brown et al., 2018; Pereira et al., 2018). As the first lymphoid organ that encounters tumor-associated antigens, TDLNs can generate both anti-tumor and pro-tumor immunity. The mechanism remains unclear but appears to be largely dependent on the stage of tumor growth (Turley et al., 2010; Swartz and Lund, 2012). Recent reports show that immune checkpoint therapy targeting the TDLN can augment systemic anti-tumor immunity, indicating the importance of cancer immunity in the TDLN during tumor growth and therapies (Dammeijer et al., 2020; Francis et al., 2020). Understanding how tumor growth regulates cancer immunity in the TDLN will largely benefit the identification of new targets to improve cancer immunotherapy.

Both T cells and B cells are primed in the TDLNs. The role of T cells is well-recognized, as it has been shown that tumor-associated DCs are immune suppressive and induce tolerogenic T cells (Veglia and Gabrilovich, 2017). Promoting anti-tumor T cell activity is a prime target for cancer immunotherapy (Hodi et al., 2010; Chen and Han, 2015; Wei et al., 2017). On the other hand, B cell cancer immunity is poorly understood and remains highly controversial (Nelson, 2010; Yuen et al., 2016; Wouters and Nelson, 2018; Sharonov et al., 2020). B cell infiltration in tumors is relatively minor compared to T cells (Bindea et al., 2013; Chevrier et al., 2017). In clinical studies, B cell accumulation is often observed in the tumor-associated tertiary lymphoid structure, which is associated with favorable prognoses and better outcomes from cancer therapies (Kroeger et al., 2016; Cabrita et al., 2020; Helmink et al., 2020; Petitprez et al., 2020). However, antibodies specific to different tumor antigens may be associated with the contradictory results of a favorable or poor prognosis (Reuschenbach et al., 2009). B cell-deficient mice inhibit tumor growth through increased cytotoxic CD8 T cell and reduced regulatory T cell populations in several tumor models, such as EL4 thymoma, MC38 colon carcinoma, and EMT-6 breast carcinoma (Shah et al., 2005; Tadmor et al., 2011; Zhang et al., 2013). In a fibrosarcoma model, depleting B cells before tumor implantation accelerates tumor growth while depleting B cells after tumor establishment suppresses tumor growth, suggesting that B cells may contribute to pro-tumor or anti-tumor immunity depending on the stage of tumor growth (Maglioco et al., 2017).

Because of these uncertainties, to better understand how to target B cells for immunotherapy, it is crucial to investigate how B cells participate in cancer immune regulation during the early stages of tumor growth. The germinal center (GC) is a microstructure induced in secondary lymphoid organs after antigen-specific B cells are activated. With the support of T follicular helper cells (Tfh) and follicular dendritic cells (FDC), antigen-specific B cells undergo rapid proliferation, somatic hypermutation, affinity selection, and isotype switching in the GCs. Eventually, they develop into high-affinity antibody-secreting plasma cells and memory B cells. Tumor-associated antigen accumulation in the GCs is observed in animal models and clinical samples, indicating an antigen-specific B cell activation and maturation (Mariani-Costantini et al., 1991; Rodriguez-Pinto et al., 2009; Moalli et al., 2015). Antigen-specific B cell maturation in GCs primarily depends on antigens presented by the follicular dendritic cells (FDCs) in the GCs. Typically, there are two major routes of antigen trafficking to FDCs (Gonzalez et al., 2011; Gray and Cyster, 2012). First, CD169^+^ subcapsular sinus (SCS) macrophages capture and relay antigen complexes to noncognate B cells (Phan et al., 2009), and those noncognate B cells can subsequently deposit the acquired antigens to the FDCs (Gonzalez et al., 2011; Heesters et al., 2016). Second, LN conduits allow small molecular weight lymph-borne antigen delivery to B cells or FDCs (Roozendaal et al., 2009; Gonzalez et al., 2011; Heesters et al., 2016). In this study, using green fluorescent protein (GFP) labeled E0771 breast cancer (E0771-GFP) and B16F10 melanoma (B16F10-GFP) cell lines in syngeneic C57BL/6 mice, we characterize the ability of FDCs in the GCs to sequester tumor-associated antigens (GFP) in the TDLN. We found that tumor growth reconstructs the TDLN microenvironment by interrupting the SCS macrophage layer and inducing LN conduits to promote tumor-associated GFP accumulation in the GCs. Regional depletion of SCS macrophages can promote GC formation and tumor growth. Our studies suggest that TDLN reconstruction promotes tumor-associated antigen delivery to the FDCs to favor antigen-specific B cell maturation during tumor growth**.**

## Materials and Methods

### Animals

C57BL/6 mice were purchased from Jackson Laboratory and were maintained at the Health Sciences Animal Resource Center at the University of Calgary. All experiments were performed using 6–8 weeks old female mice. All animal protocols were reviewed and approved by the University of Calgary Animal Care and Ethics Committee and conformed to the Canadian Council on Animal Care guidelines.

### Tumor Cell Culture and Implantation

E0771 is a C57BL/6 syngeneic, triple-negative, spontaneously developing medullary breast adenocarcinoma purchased from CH3 Biosystems. E0771 cells were cultured at 37°C and 5% CO_2_ in a T75 flask. High glucose (4500 mg/L) Dulbecco’s Modified Eagle’s Medium was supplemented with 10% heat-inactivated FBS, then filtered through a 0.22 µm filter. Cells were harvested at 80–90% confluency for use. E0771-GFP and B16F10-GFP cell lines were gifts from Dr. Timothy Padera’s lab. Tumor cells were infected with Adeno-associated virus (AAV) constructs encoding the GFP reporter gene. Mycoplasma was tested routinely for all the cell lines used. Cells were removed from the flask using trypsin then resuspended in PBS for injection. The fur surrounding the fourth mammary fat pad was removed using a hair removal cream, then 1.0 × 10^6^ cells were injected into the fourth mammary fat pad on the right side of the mice. Tumors were allowed to establish and grow for 7 days before measurements were taken.

### Clodronate Liposome Treatment

Control and clodronate liposomes were purchased from Liposoma Research. The right flank was shaved, and 50 µl of control liposomes (containing PBS) (PBS-L) or clodronate liposomes (CLL) was intradermally injected 7 days prior to tumor implantation. Liposomes were then injected weekly to maintain macrophage depletion. For systemic CLL treatment, 200 µl of control PBS-L or CLL were injected intraperitoneally (i.p.) 7 days prior to tumor implantation and then weekly to maintain macrophage depletion.

### Collection of Tissues

TDLNs were excised from euthanized mice, and the contralateral inguinal LNs were collected as control, non-tumor draining LN. For some studies, tissues were directly embedded in O.C.T. for cryosection. In other studies that needed to preserve GFP fluorescence, samples were fixed in 4% formalin for 1 hour before being embedded in O.C.T. for cryosection. Blood was collected using the optical bleed method.

### Immunofluorescent Staining and Imaging

LN cryosections are stained using a standard protocol. Frozen sections are blocked with 5% mouse serum for 1 hour. Samples were then incubated overnight at 4°C with primary antibodies. After three washes in PBS, samples were incubated with fluorescent probe-conjugated secondary antibodies (Jackson ImmunoResearch) for 1 hour. The primary antibodies used are rabbit anti-Lyve-1 (Clone aa24-228), purified rat anti-mouse CD169 (Clone 3D6.112), anti-Collagen I antibody (Clone EPR22894-89), purified anti-mouse/human GL7 antigen (Clone GL7), Lectin from *Arachis hypogaea* (PNA) (SIGMA, L6135-1 MG), purified rat anti-mouse CD45R (Clone RA3-6B2), purified rat anti-mouse CD21/35 (Clone 7E9), purified rabbit anti-mouse Ki67 (Clone SP6), purified rabbit anti-mouse elastin (Clone EPR20603).

Slides were imaged using an SP8 confocal microscope (Leica). The objectives used were 20× air and 63× oil, depending on the study. LN sections were collected at the center area of the LNs to ensure MS, SCS, and LN parenchyma were representative. Whole mount lymph node samples were imaged using an SP8 multiphoton microscope (Leica). The objective used was 25× water.

### Image Analysis

Image analysis was done on the LASX and ImageJ software. Depth measurements were measured using the measurement feature on the LASX program for the SP8 confocal microscope (Leica). Multiple measurements were taken per LN, per condition. Collagen density in B cell zones was evaluated on the ImageJ software. Images of the B cell zone at 63× oil objective were divided into two separate channels: DAPI and Collagen I^+^. The image was limited by approximately tracing the B cell zone, then utilizing the “Clear Outside” function on ImageJ, removing signal found outside of the traced B cell zone. Using the “Adjust Threshold” function on ImageJ, the Collagen I^+^ and DAPI signals are targeted for measurement. Subsequently, the “Analyze Particles” function provides a readout of the “%Area”, which represents what fraction of the total image is positive signal (Collagen I^+^ and DAPI^+^). To make the Collagen I^+^ signal relative to the DAPI^+^ signal and exclude the empty space from calculation, Collagen I^+^ signal is divided by DAPI^+^ signal, giving the percentage of Collagen I^+^ within the DAPI^+^ B cell zone.

### ELISA

Buffers, substrates, and standards were prepared in accordance with the Cayman Chemical Mouse IgG ELISA Kit pre-assay preparation (Cayman Chemical 501240). Complete ELISA procedure was conducted according to the Cayman Chemical Mouse IgG ELISA Kit instructions.

### Flow Cytometry

LNs were manually dissociated using a plunger over a 40 μm cell strainer, then resuspended in FACS buffer before briefly blocking with purified rat anti-mouse CD16/CD32 (mouse Fc Block) (Clone Ab93 (RUO)) for 5 minutes at room temperature. Cells were then stained with an antibody master mix for 40 min at 4°C. Samples were washed, then fixed in 2% formalin in FACS buffer. Samples were recorded using a BD FACSCANTO II machine and results were analyzed using the FlowJo software. Antibodies for flow cytometry were: CD169 (FITC, PerCP Cy5.5, APC: Clone 3D6.112), CD8 (FITC, PE: Clone 53-6.7), F4/80 (PE, APC: Clone BM8), CD19 (PE, APC: Clone 6D5), CD4 (PerCP Cy5.5: Clone RM4-5), CD45 (PerCP Cy5.5, PE Cy7, APC Cy7: Clone 30-F11).

## Results

### Tumor-associated GFP Accumulated in the Germinal Centers of TDLN

**FIGURE 1:**
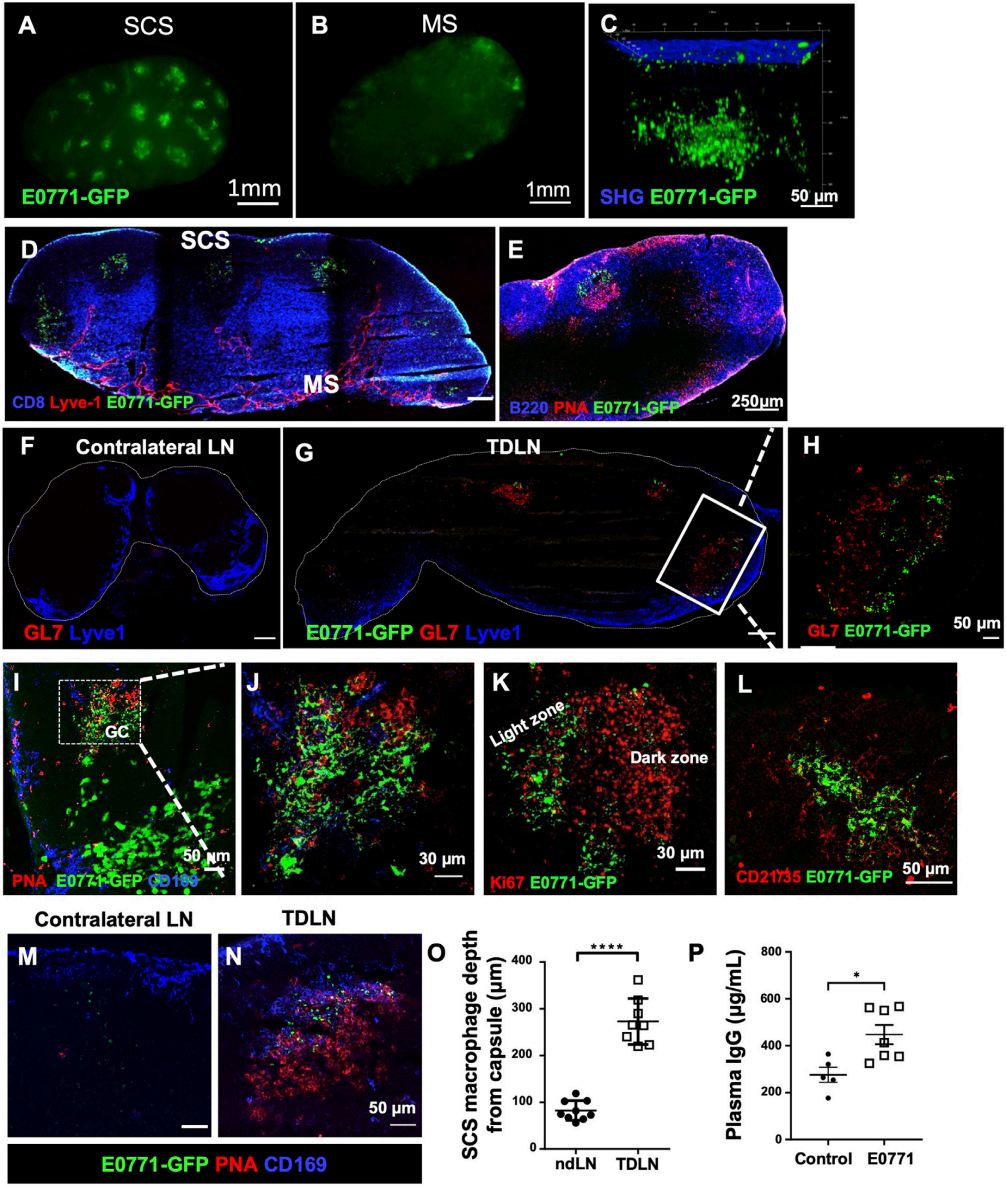
Tumor-associated antigens accumulate at the germinal centers (GCs). **(A,B)** Whole-mount images by a stereotype microscope show the subcapsular sinus (SCS) side **(A)** and the medullary sinus (MS) side **(B)** of day 21 E0771-GFP TDLN. *n* = 5. **(C)** Multiphoton microscopic image shows GFP^+^ tumor-associated antigen underlying the LN capsule, visualized by second-harmonic generation (SHG). *n* = 5. **(D–J)**. GFP accumulates in the B cell zone of TDLN, but not in the ndLN. GCs were highlighted by PNA and GL7. **(K)** GFP localized in the light zone of the GC as the dark zone was indicated by Ki67 staining. **(L)** GFP was deposited in the CD21/35^+^ follicular dendritic cells (FDCs) of the TDLNs. *n* = 5. **(M–O)** CD169 staining show the location of SCS macrophages in the TDLN. Image quantification of CD169^+^ macrophages staining showed them significantly deeper in the LN cortex. Data is mean ± SEM. ****p < 0.0001. Mann-Whitney U test. Images are representative of *n* = 8-9 per group, repeated three times. **(P)** E0771 mice show significantly higher plasma IgG compared to control wild-type mice. Data is mean ± SEM. *p < 0.05. Mann-Whitney U test (*n* = 5–7 per group, in two separated studies).

E0771-GFP cells (1 × 10^6^) were injected into the fourth mammary fat pad, and the tumor-draining inguinal LNs (TDLNs) were collected 21 days post-tumor implantation to determine where metastatic tumor cells and tumor-associated GFP were located. With whole-mount LN imaging using a stereotype fluorescent microscope, GFP fluorescence was detected close to the SCS of the TDLN (Figures 1A,B). Using 3D reconstructed images with multiphoton microscopy, we found GFP was clustered approximately 100–200 μm under the LN capsule (indicated by second harmonic generation, SHG signal) (Figure 1C). Immunofluorescent (IF) staining using cryosections showed that the GFP^+^ signal was consistently detected in the B cell zone within PNA^+^ and GL7^+^ GCs (Figures 1D–H). Unlike metastatic tumor cells that showed a large size and smooth surface, GFP^+^ clusters in the GCs appeared fragmented (Figures 1I,J). Within the B cell zone, GFP^+^ clusters were localized in the light zone of GCs indicated by its distinct location relative to Ki67 (indicative of the proliferating dark zone) and in CD21/35^+^ follicular dendritic cells (FDCs) (Figures 1K,L). CD169^+^ SCS macrophages appeared to relocate deeper into the B cell zone and were closely associated with GFP in the GCs (Figures 1M–O). GCs are the structures that support antigen-specific B cell maturation and high-affinity antibody production. We measured antibody concentration in the blood circulation and found increased plasma IgG levels at day 21 post-tumor implantation (Figure 1P). Cumulatively, these observations show that GCs were induced in the TDLN, tumor-associated GFP was deposited in the FDCs of GCs, and antibody production was increased during tumor growth.

### Tumor-associated GFP Accumulation in the GCs of the TDLN Depends on Tumor Growth

**FIGURE 2:**
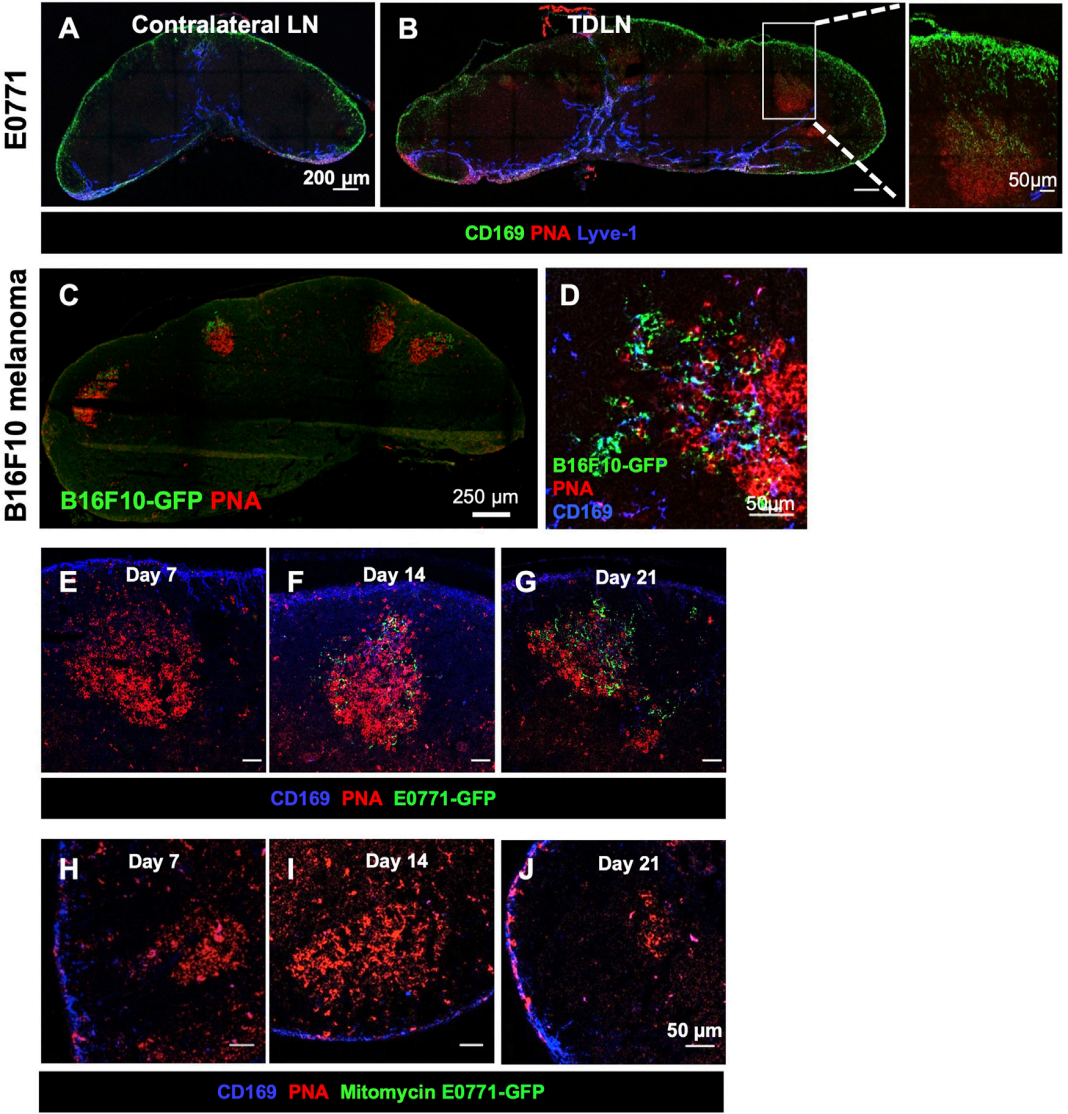
Tumor-associated antigen accumulation depends on a growing tumor. **(A,B)** GC formation and SCS macrophage colocalization occurs in E0771 TDLN. Images are representative of *n* = 5 per group, in two separated studies. **(C,D)** B16F10-GFP melanoma show tumor-associated antigen (GFP) accumulation in the GCs with CD169^+^ SCS macrophage in the TDLNs. *n* = 5. **(E–G)** GC and GFP in the TDLN at different time points post-E0771-GFP implantation. **(H–J)** GC and GFP in the LN when injected with 10^7^ mitomycin-treated E0771-GFP cells. IF images are a representative of *n* = 5 per group, in two separated studies.

GCs are typically induced after foreign antigens activate B cells. To exclude the possibility that GFP is necessary to stimulate GC formation, we implanted E0771 tumor cells and collected the TDLNs on day 21 post-tumor implantation. GCs were consistently detected in the TDLN, and the CD169^+^ SCS macrophages appeared to migrate from the SCS to the GCs, which is consistent with our results using the GFP-labeled E0771 cell line (Figures 2A,B). To determine if GC formation and tumor-associated GFP accumulation in GCs is unique to the E0771 breast cancer cell line, we conducted the same experiment with a B16F10 melanoma cell line (B16F10-GFP). GFP^+^ clusters were found in the GCs in the TDLN (Figures 2C,D). Thus, both E0771 and B16F10 tumor growth resulted in the accumulation of tumor-associated GFP in the GCs of the TDLNs.

To determine when tumor-associated GFP started accumulating in the FDCs in the TDLN, we collected TDLNs at day 7, 14, and 21 days post-tumor implantation. PNA^+^ GCs were detected as early as 7 days after implantation. GFP signal in the GCs was consistently detected 14 days post-implantation and increased over time (Figures 2E–G). To exclude the possibility that GFP fluorescence was too weak to be detected in the day 7 TDLN, we used an anti-GFP antibody to increase the sensitivity. Results showed that the anti-GFP signal was highly colocalized with GFP in the day 21 TDLN. However, we could not detect any anti-GFP signal in the day 7 TDLN (Supplementary Figure S1). These results showed that tumor-associated GFP preferentially accumulated in the GCs after GCs were formed in the TDLNs.

Next, we injected mitomycin-treated E0771-GFP cells (1 × 10^7^) to determine if active tumor growth is necessary for GC formation. The mitomycin-induced apoptotic tumor cells were sufficient to induce GC formation by day 7, but GCs were dissociated by 21 days post-implantation. No GFP signal was detected in any of these LNs (Figures 2H–J). Thus, the duration of GCs in the TDLN and the accumulation of tumor-associated GFP in the GC were dependent on active tumor growth.

### Tumor Growth Promotes Conduit Expansion and Allows Soluble Antigen Entry Into the GCs

**FIGURE 3:**
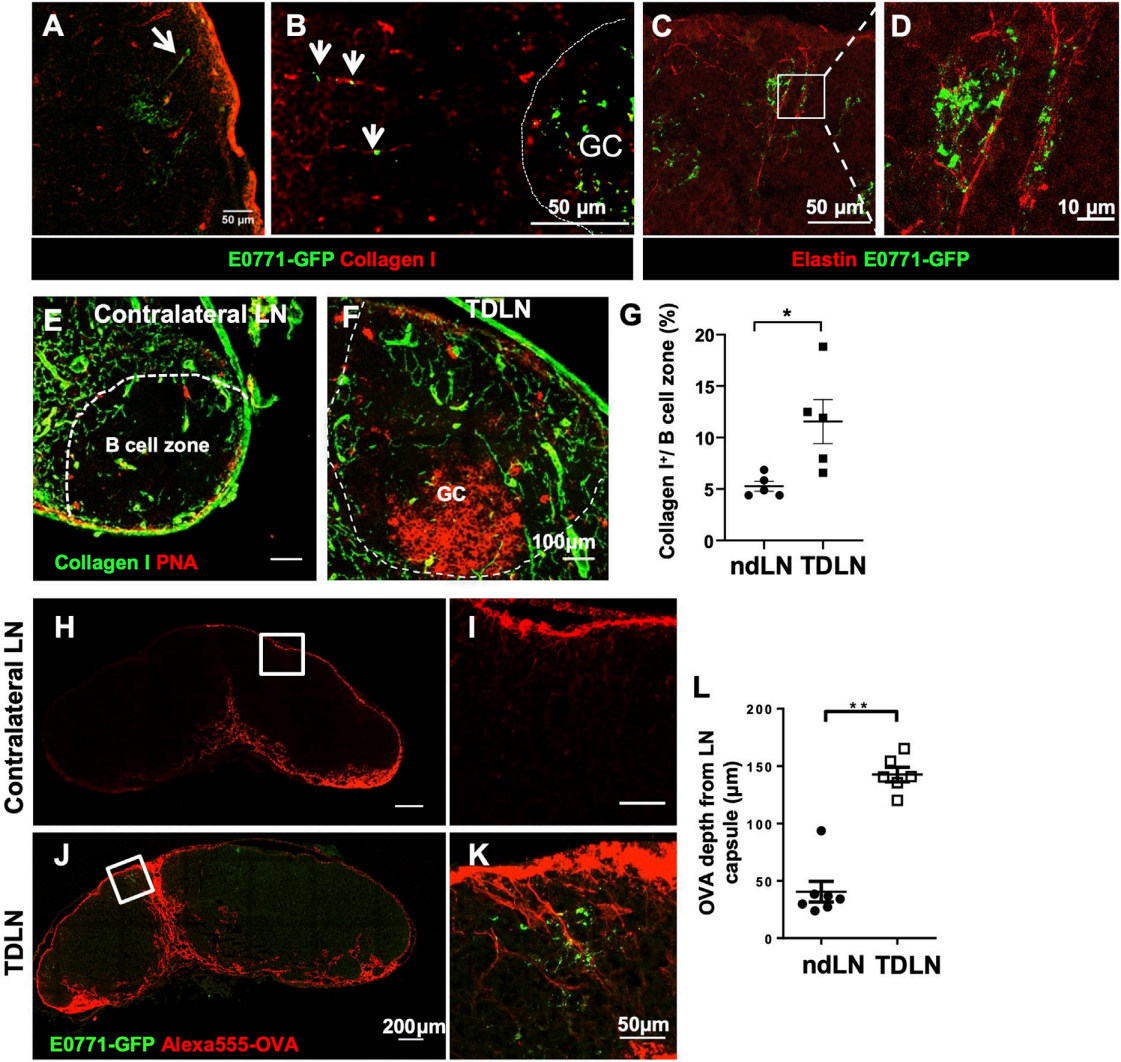
Tumor growth reconstructs conduits to facilitate small antigen entry into the B cell zone and GCs. **(A–D)** Outside of the GCs, GFP was associated with Collagen I^+^ **(A,B)** and Elastin^+^ **(C,D)** conduits. *n* = 5. **(E–G)** Collagen I^+^ conduits in the B cell zone were more abundant in the TDLN than the contralateral ndLN. Images are representative of *n* = 5 per group, repeated twice. Quantification is the average of all B cell zones per lymph node section per mouse. Data is mean ± SEM. *p < 0.05. Unpaired, parametric student’s *t*-test. **(H–L)** Alexa555-conjugated ovalbumin (OVA) penetrated deeper into the LN cortex toward GFP clusters in the GCs in the TDLNs. Data is mean ± SEM. **p < 0.01. Mann-Whitney U test. Images are a representative of *n* = 5**–**7 per group, in two separated experiments. Image quantification is the average of three measurements per lymph node section per mouse.

B cells can directly acquire lymph-borne antigens traveling along the LN conduits and indirectly acquire antigens from SCS macrophages (Gonzalez et al., 2011; Gray and Cyster, 2012). LN conduits are a specialized network composed of a bundle of collagen fibers, elastin, and other extracellular matrix proteins. Using immunofluorescent staining, we found that while GFP is clustered with FDCs in the GCs, GFP outside the GCs was closely associated with LN conduits labeled by collagen I (Figures 3A,B) and elastin (Figures 3C,D).

It has been previously established that conduits are sparse in the B cell zone of naïve LNs (Bajenoff and Germain, 2009). In this study, we found an expansion of the conduit network into the B cell zone of the TDLN (Figures 3E–G). Since conduits only traffic small molecular weight antigens, we injected 20 μg Alexa555 fluorescently-labeled OVA (Alexa555-OVA) at the flanks near the contralateral non-draining LNs and the TDLNs and collected LNs 2 h later. In LN cryosectitons, Alexa555-OVA drained deeper into the LN parenchyma and some preferentially drained through the conduits towards the area where the Tumor-associated GFP was clustered (Figures 3H–L). These results showed that LN conduits were reconstructed to improve soluble antigen trafficking toward the GCs during tumor growth.

### Regional Delivery of Clodronate Liposome (CLL) Specifically Depletes CD169^+^ SCS Macrophages in the TDLN

**FIGURE 4:**
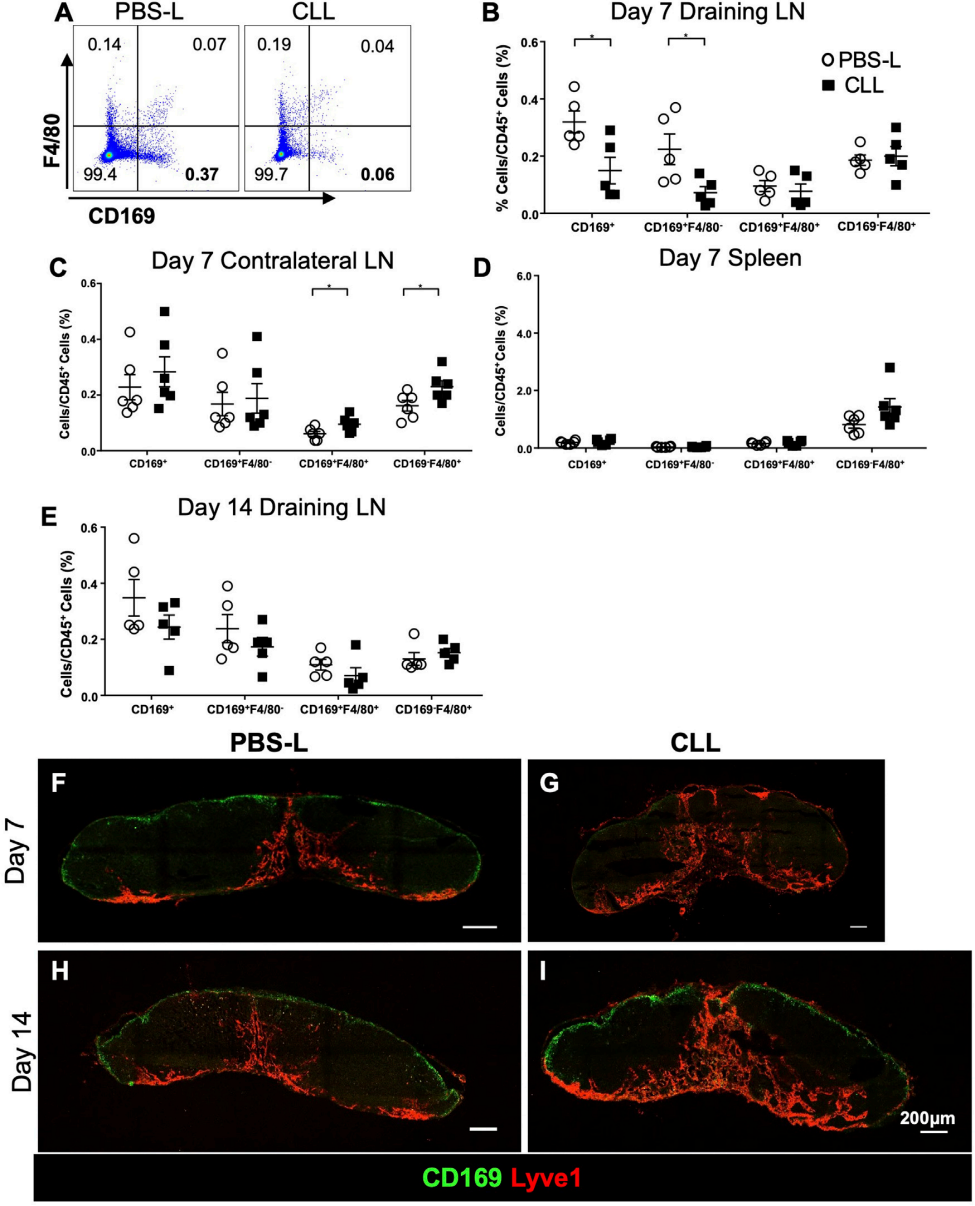
Regional CLL delivery specifically depletes SCS macrophages in the draining lymph node 7 days post-injection and are recovered 14 days post-injection. **(A–D)** Flow cytometry of the lymph nodes and spleens demonstrate the specific depletion of CD169^+^F4/80^−^ LN SCS macrophages 7 days post-intradermal flank injection (*n* = 5**–**6 per group, repeated twice). Data is mean ± SEM. *p < 0.05. Unpaired, parametric student’s *t*-test. **(E)** Flow cytometry of the draining lymph node 14 days post-intradermal CLL flank injection (*n* = 5**–**6 per group, in two separated experiments). **(F–I)** IF staining shows CLL flank injection depleted the CD169^+^ SCS macrophage layer in the draining lymph node 7 days post-intradermal injection. The macrophages are recovered 14 days post-intradermal injection. Images are representative of *n* = 5 per group, in two separated experiments.

Because SCS macrophages appeared to relocate from the SCS to the GCs (Figures 1M–O), we hypothesized that SCS macrophages facilitate antigen delivery to the GCs. To test this hypothesis, we specifically depleted SCS macrophages in the TDLN via intradermal injection of clodronate liposomes (CLL) or control liposomes only containing PBS (PBS-L). SCS macrophages (CD169^+^F4/80^−^) were significantly depleted in the draining LN by day 7 post-CLL treatment, but other macrophages (F4/80^+^) were not significantly changed (Figures 4A,B). The treatment did not impact macrophage populations in the contralateral non-draining LN or spleen (Figures 4C,D). SCS macrophages were recovered 14 days after the CLL injection (Figure 4E). To better characterize SCS macrophage localization in the LN after liposome treatment, we collected LNs at day 7 and day 14 post-CLL or PBS-L treatment. By IF staining, SCS macrophages were well depleted by day 7 (Figures 4F,G) and the SCS macrophage layer was restored by day 14 post-treatment (Figures 4H,I). Therefore, regional CLL delivery specifically depleted SCS macrophages in the draining LN.

### Regional Depletion of CD169^+^ Macrophages in the TDLN Promoted Tumor Growth

**FIGURE 5:**
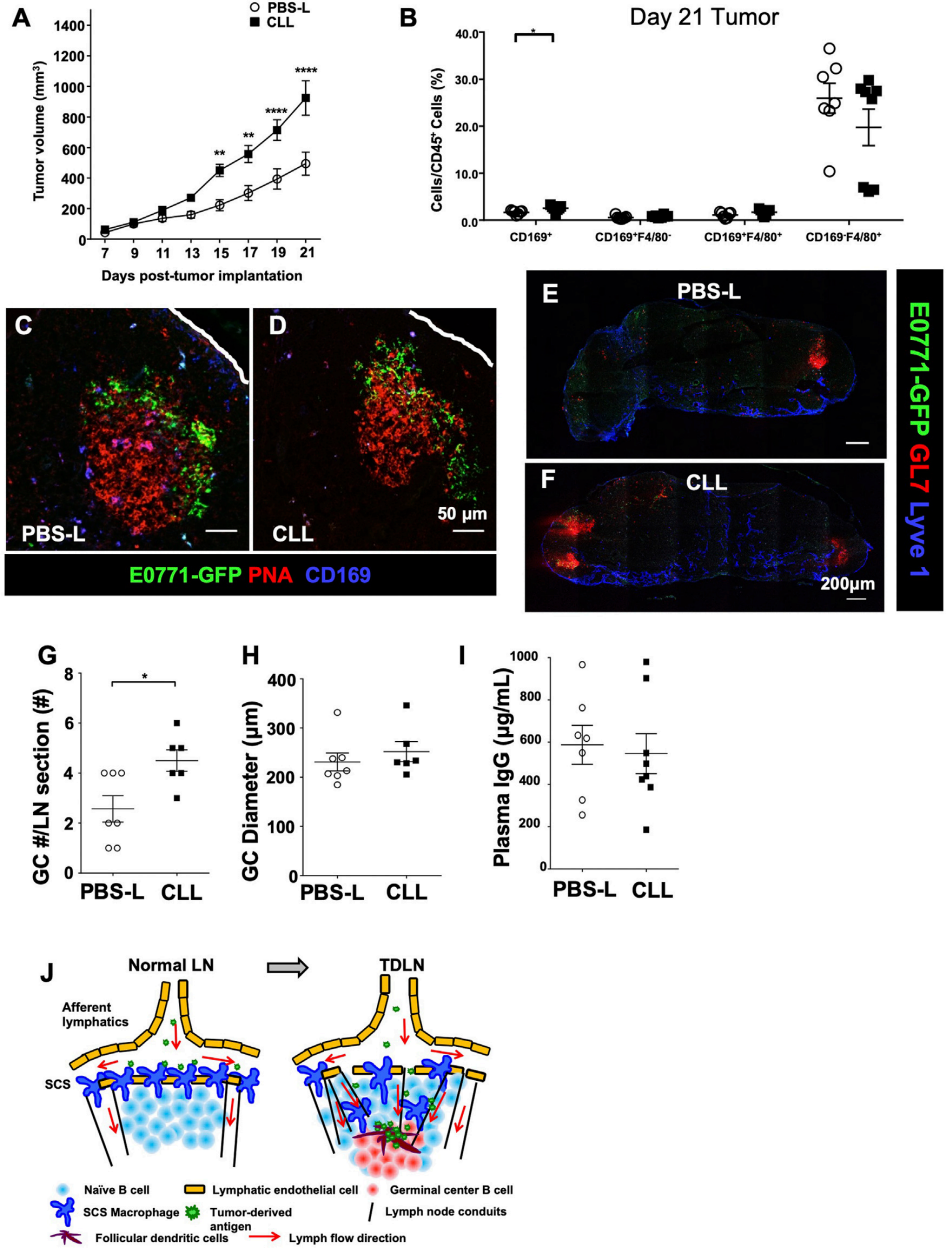
Tumor growth reconstructs CD169^+^ SCS macrophages to promote tumor-derived antigen entry into GCs. **(A)** SCS macrophage depletion with CLL significantly increases E0771 tumor growth over 21 days (*n* = 10 per group, in two separated experiments). Data is mean ± SEM. **p < 0.01, ****p < 0.0001. Two-way ANOVA with Sidak’s multiple comparisons test. **(B)** Flow cytometry of E0771-GFP tumor macrophage populations demonstrates no depletion of tumor-associated macrophages from CLL treatment (*n* = 5-6 per group, in two separated experiments). Data is mean ± SEM. *p < 0.05. Unpaired, parametric student’s *t*-test. **(C,D)** GFP in GCs remains unchanged between control PBS-L and CLL-treated mice. Images are representative of *n* = 5 per group, in two separated experiments. **(E–H)** The number of GCs was increases per LN in CLL-treated mice compared to control PBS-L-treated mice, but diameter of the GCs remains similar. Images are representative of *n* = 6-7 per group, in two separated experiments. Data is mean ± SEM. *p < 0.05. Mann-Whitney U test. **(I)** The amount of plasma IgG did not change between PBS-L and CLL-treated E0771-GFP mice (*n* = 7-8 per group, in two separated experiments). Data is mean ± SEM. Mann-Whitney U test. **(J)** Schematic model of TDLN reconstruction and antigen delivery to GCs. Tumor growth induces TDLN conduit expansion in the B cell zone and reduces SCS macrophage layer, both facilitate tumor-associated antigen delivery to GCs.

Based on the SCS macrophage depletion duration (Figure 4), we injected PBS-L or CLL 7 days prior to the tumor implantation, at the time of the implantation, and weekly after the implantation to maintain CD169^+^ macrophage depletion throughout tumor growth. PBS-L or CLL was injected in the flank to avoid depleting macrophages in the primary tumor, and the tumor was implanted in the fourth mammary fat pad. E0771-GFP tumor growth was increased in mice treated with CLL compared to the mice treated with PBS-L (Figure 5A). Tumor and TDLNs were collected on day 21 post-tumor implantation. Flow cytometry analysis confirmed that tumor-associated macrophages were not depleted by the liposome treatment (Figure 5B). GFP in the GCs was detected in both PBS-L and CLL-treated mice, and the distribution pattern of GFP in the GCs was comparable between PBS-L and CLL groups (Figures 5C,D). Using anti-GL7 and anti-Lyve1 (lymphatic endothelial cell) antibodies, we found a higher number of GCs per LN section in the CLL-treated mice compared to the PBS-L treated mice, but the diameters of the GCs were similar in both conditions (Figures 5E–H). Together, these results show that the reduced SCS macrophage layer enhances GC formation in the TDLN.

Next, we compared plasma IgG levels at day 21 post-E0771-GFP tumor implantation between PBS-L and CLL-treated mice. To our surprise, IgG levels were comparable between PBS-L and CLL-treated mice (Figure 5I). We also compared immune cell components in the tumors at day 21 post-E0771-GFP implantation and found that CD8^+^ T cell infiltration was reduced in the CLL-treated mice compared to PBS-L treated mice. CD4^+^ and CD19^+^ cells in the tumor are comparable (Supplementary Figure S2).

## Discussion

During tumor growth, TDLNs are exposed to a variety of tumor-associated antigens, such as metastatic/apoptotic tumor cells, extracellular vesicles, necrotic debris, and soluble factors. Normally, tumor-associated antigens are acquired by antigen-presenting cells (APCs), such as dendritic cells and macrophages. APCs process and present the acquired antigens to T cells in the TDLN. Our studies show that GCs are induced in the TDLNs, and GFP, a representative tumor-associated antigen from our tumor model, preferentially accumulates in CD21/35^+^ FDCs in the GCs. FDCs can sequester and present antigens in the GCs for high antigen-affinity B cell selection and maturation into antibody-secreting plasma cells (Gonzalez et al., 2011; Heesters et al., 2016). The increased plasma IgG level further confirms the induction of humoral immunity during E0771 tumor growth. Our results showed that by promoting LN conduit expansion in the B cell zone and reducing the SCS macrophage layer, TDLN reconstruction favors tumor-associated antigen delivery to the FDCs in the GCs and promoted humoral responses (Figure 5J).

Our study showed that GCs were formed as early as 7 days post-tumor implantation. Since GC formation was consistent when using both GFP-labeled and unlabelled E0771 tumor cells, we excluded the possibility that GCs are induced by the foreign antigen GFP. The endogenous tumor-associated antigens were sufficient to induce GC formation in the TDLN. During early stages of tumor growth, GFP as well as endogenous tumor-associated antigens can be acquired and processed by APCs, and then presented to T cells. Some antigen-specific CD4^+^ T cells can be primed into Tfh. Antigen-specific B cells can also acquire tumor-associated antigens and process them. The activated antigen-specific B cells then interact with the activated Tfh to initiate the GCs in the TDLN. Since the GFP fluorescent signal can only be seen in its native form, the processed GFP cannot be detected by fluorescent imaging an early stage of tumor growth. This also interprets why GFP was not detected in other areas in the TDLN. Later, after GCs are formed, GFP accumulation was mostly found in the FDCs in the light zone of the GC. This step is most likely because more GFP is released from the growing tumor in various forms and GFP can be preserved in its native form to be detected in the FDCs by IF imaging. Our study showed that mitomycin-treated tumor cells (apoptotic tumor cells) could release tumor-associated antigens to induce GCs in the LN. But GFP fluorescent signal in the GCs required a growing tumor to continuously release GFP. How GFP in the TDLN after GC formation can be preserved in its native form and be detected by its fluorescent signal is not clear. We observed GFP in GCs in all TDLNs but rarely found metastatic tumor cells in the TDLNs. In those few TDLNs with tumor cells, GFP^+^ tumor cells are located very close to the SCS. Tumor cells were large, had a smooth surface, and some of them were Ki67^+^ proliferating cells (Supplementary Figure S3). Thus, tumor-associated GFP in the GCs was not dependent on metastatic tumor cells in the TDLNs. FDCs are known to be able to retain antigens in their native form or in immunocomplexes for a relatively long time for B cell maturation (Phan et al., 2009; Gonzalez et al., 2011; Heesters et al., 2016). Because GFP was detected after GC was formed, one possibility is GFP may form immune complexes with GFP specific antibodies produced in GCs in the TDLN. Another possibility is GFP may be contained in tumor-derived exosomes and preserved in its native form (Pucci et al., 2016). GFP is retained in FDCs after GC formation, indicating endogenous tumor-associated antigens can also be preserved in the FDCs for further B cell selection to produce high-affinity tumor specific antibodies.

LN conduits, a pathway for soluble antigen entry into the LN parenchyma, are composed of a core of collagen fibers and wrapped by fibroblastic reticular cells. Conduits are usually sparse in the B cell zone, and our most notable finding is the increased collagen I^+^ conduits within the B cell zones of the TDLN. We observed that the GFP signals outside the GCs are associated with conduits. Using Alexa555-OVA as a tracer, OVA could drain deeper into the LN towards the GCs, which indicated that the reconstructed conduits promote small molecular weight antigen entry into the GCs in TDLN. The induction of LN conduits unveils a previously unrecognized route of tumor-associated antigen delivery into the GCs in the TDLN to improve antigen-specific B cell selection and maturation (Figure 5J).

SCS macrophages are well known to acquire lymph-borne antigens, but the situation is more complicated in the context of tumor growth. Previous studies have shown that radiation-induced apoptotic tumor cells are acquired and presented to activate CD8^+^ T cells and prevent tumor growth (Asano et al., 2018). Clinically, a higher density of SCS macrophages is correlated with improved patient survival and a more favorable prognosis in melanoma, colorectal carcinoma, and hepatocellular carcinoma cases (Ohnishi et al., 2013; Saito et al., 2015; Zhang et al., 2016; Asano et al., 2018). In these conditions, the higher density of SCS macrophages promotes anti-tumor CD8^+^ T cell responses to inhibit tumor growth.

In contrast, the role of SCS macrophages in regulating B cells in the TDLN remains controversial. It is known that when large molecular weight antigens, such as immune complexes, enter the LN, SCS macrophages capture and relay them to B cells via an antigen unspecific, complement receptor 3 mediated process (Phan et al., 2009). Eventually, the non-cognate B cells deposit the acquired antigens onto FDCs for long-term storage, contributing to cognate B cell activation and affinity maturation in the GCs (Gonzalez et al., 2011; Heesters et al., 2016). SCS macrophages play a critical barrier function in the LN as they capture lymph-borne microbes and prevent the spread of microbes in infectious diseases (Iannacone et al., 2010; Kastenmuller et al., 2012; Moseman et al., 2012; Farrell et al., 2015). In our study, E0771-GFP tumor growth induced TDLN expansion (Supplementary Figures S4A,B). Using flow cytometry, we found that the proportion of the SCS macrophages was significantly reduced. However, the number of SCS macrophages was similar between the contralateral and the TDLN (Supplementary Figure S4C,D). SCS macrophages relocated deeper into the LN, which resulted in the reduced SCS macrophage layer. Some of the GFP signals were closely associated with CD169^+^ staining (Figures 1M–O).

It has been reported that the disruption of SCS macrophages during inflammation impairs B cell responses to secondary infection (Gaya et al., 2015). With the reconstruction of the TDLN and the reduction in the SCS macrophage layer in the TDLN, we sought to determine how the tumor-induced immune response would be affected with an artificial reduction in the SCS macrophage layer. To our surprise, in the TDLN of E0771-GFP-bearing mice, our study showed that regional depletion of SCS macrophages promoted GC formation and tumor growth (Figure 5). These results are consistent with the B16F10 melanoma model, in which regional depletion of the SCS macrophages permitted the entry of tumor-derived vesicles into the B cell zone, enhancing high-affinity antibody production and promoting melanoma growth (Pucci et al., 2016). In contrast, 4T1 breast cancer induces the expansion of SCS macrophages in TDLN. Regional depletion of SCS macrophages increases distant metastasis with no impact on primary tumor growth (Tacconi et al., 2021).

In a different published study, the systemic injection of CLL can also deplete LN SCS macrophages, but it also abrogates tumor-associated antigen accumulation in the GCs (Moalli et al., 2015). We also compared E0771-GFP tumor growth after administration of PBS-L or CLL. While CLL depleted SCS macrophages in the TDLN, it did not significantly impact tumor growth (Supplementary Figure S5). Our results indicated that regional and systemic drug delivery to the TDLN may have different impacts on the immune regulation in the TDLN. Therefore, the role of SCS macrophages in regulating the delivery of tumor-associated antigens and anti-tumor immunity may vary in different conditions and types of tumors.

Our study showed that B cell activation and GC formation are consequences of cancer growth. We found that E0771-GFP tumor growth was suppressed in B-cell deficient mice (μMT) (Supplementary Figure S6). Tumor antigen-specific B cells and antibodies may have pro-tumor and anti-tumor effects in different types of tumors in mouse models and clinical studies (Sharonov et al., 2020). In the B16F10 melanoma model, high-affinity antibody production promotes melanoma growth (Pucci et al., 2016). However, in a 4T1 mouse breast cancer model, high-affinity antibodies did not change tumor growth. Instead, tumor antigen-specific IgG promoted metastasis (Gu et al., 2019). In our study, depletion of SCS macrophages increased the number of GCs in the TDLN but did not increase blood IgG level. Instead, the depletion of SCS macrophages reduced CD8^+^ T cells in the tumor and promoted tumor growth. Thus, although GCs are a unique microstructure that supports antigen-specific B cell maturation into plasma cells to produce high-affinity antibodies, GCs in the TDLN can regulate tumor growth much more than producing high-affinity antibodies. Other critical events in the GCs may participate in cancer immune regulation. Recently, it was reported that in a lung adenocarcinoma mouse model, antigen-specific GC B cells interact with Tfh, which subsequently secretes IL-21 to promote anti-tumor CD8^+^ T cells (Cui et al., 2021). Therefore, it is clear that the status of B cells and the interaction between B cells and other cells in GCs (i.e., FDCs and Tfh) in the TDLN contributes to the pathogenesis of cancer. Fully elucidating how B cells participate in cancer immune regulation in the TDLN would largely contribute to current cancer immunotherapy.

## Data Availability

The raw data supporting the conclusions of this article will be made available by the authors, without undue reservation.

## Abbreviations

GC, Germinal center; LN, Lymph node; SCS, Subcapsular sinus; TDLN, Tumor-draining lymph node.
