# Supplementary material for: Tumor-Draining Lymph Node Reconstruction Promotes B Cell Activation During E0771 Mouse Breast Cancer Growth
Source: Front Pharmacol. 2022 Mar 28;13:825287. doi: 10.3389/fphar.2022.825287 (PMC8995528; doi:10.3389/fphar.2022.825287)
Supplement: Supplementary file 1 [file DataSheet1.pdf]

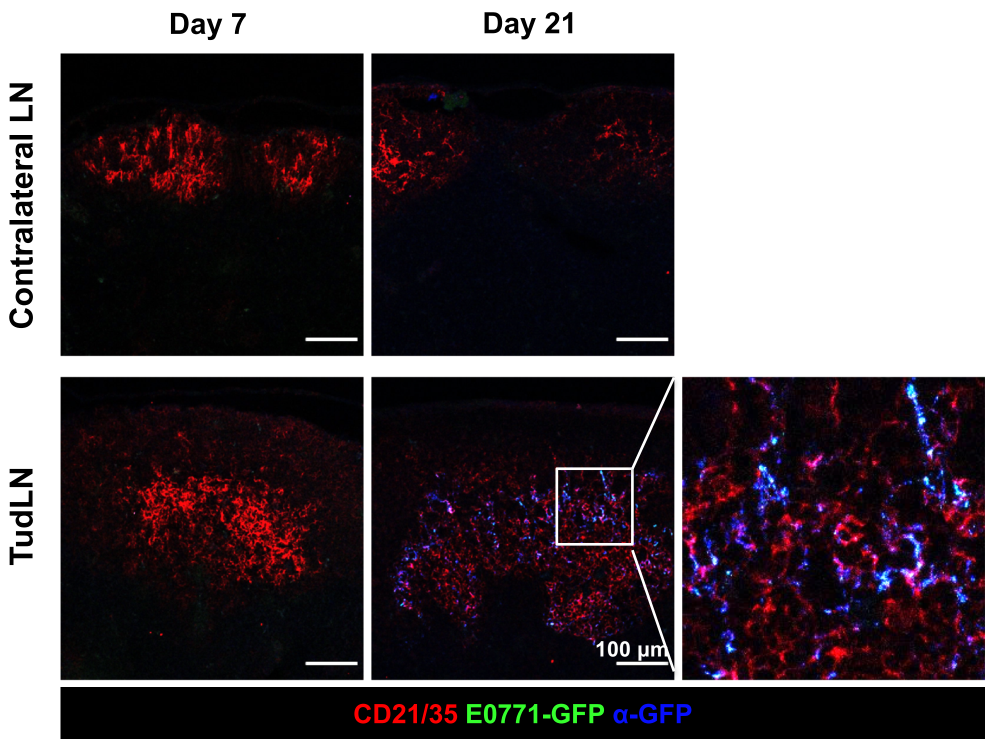

Supplemental Figure 1. Tumor-associated GFP was detected using anti-GFP antibody.

Contralateral control LNs and the TDLNs were collected on day 7 or day 21 after tumor implantation. The cryosections were stained with anti-CD21/35 (FDC marker) and anti-GFP antibody. Results showed that anti-GFP antibody signal was only co-localized with E0771-GFP in the day 21 TDLNs, indicating the specificity of the staining. We did not detect anti-GFP signal in the day 7 TDLN. These results showed that tumor-derived antigen was not accumulated in the TDLN by day 7. n=5 per group.

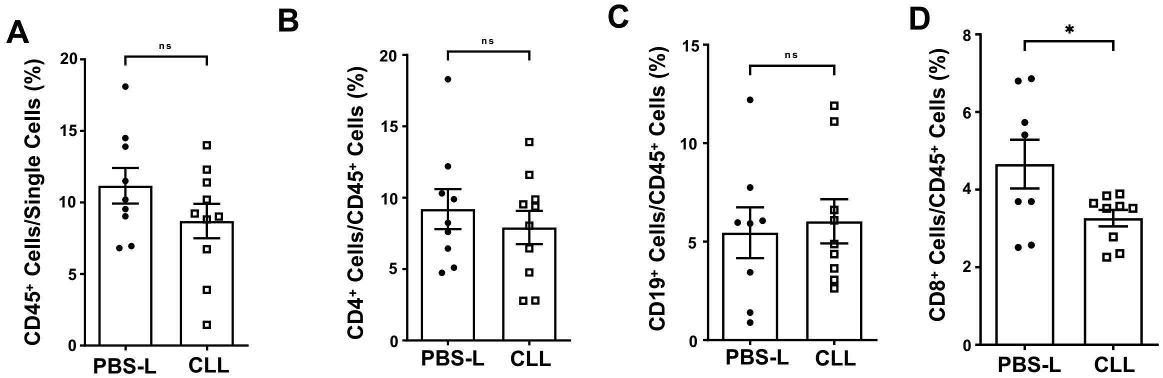

Supplemental Figure 2. Flow cytometry of E0771 tumors show that tumor-infiltrating CD8⁺ cells are decreased in CLL-treated mice 21 days post-E0771-GFP tumor implantation. A. CD45+ cells**; B.** CD4⁺ cells**; C.** CD19⁺ cells; **D.** CD8⁺ cells. (n=8-10 per group, repeated twice). *$p < 0.05$. Unpaired, parametric student's t-test.

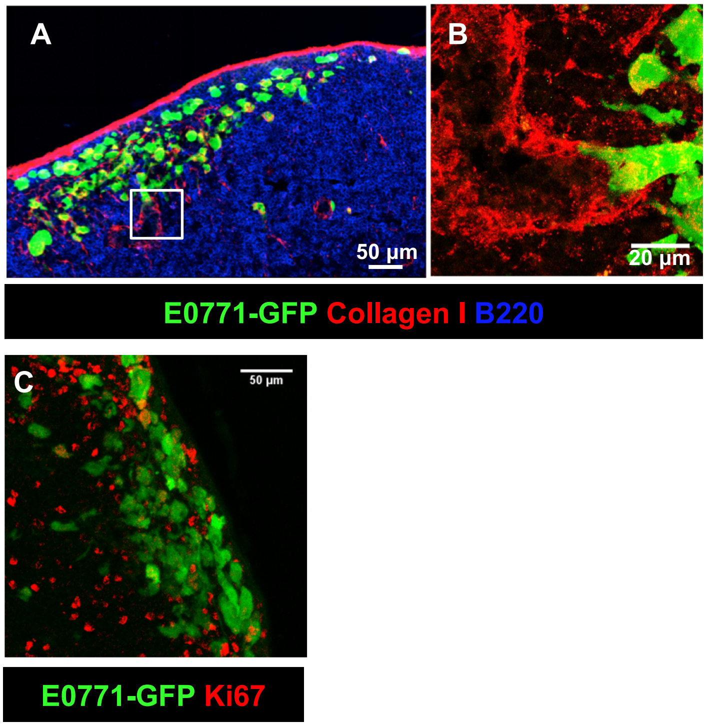

Supplemental Figure 3. GFP$^+$ E0771 metastatic tumor cells were located under the SCS. A, B. GFP$^+$ E0771 tumor cells can be found in close proximity to Collagen I$^+$ blood vessels in the tumor-draining lymph node; **C.** Some GFP$^+$ E0771 tumor cells express Ki67. Metastatic tumor cells were only found in 2-3 TDLNs from different sets studies.

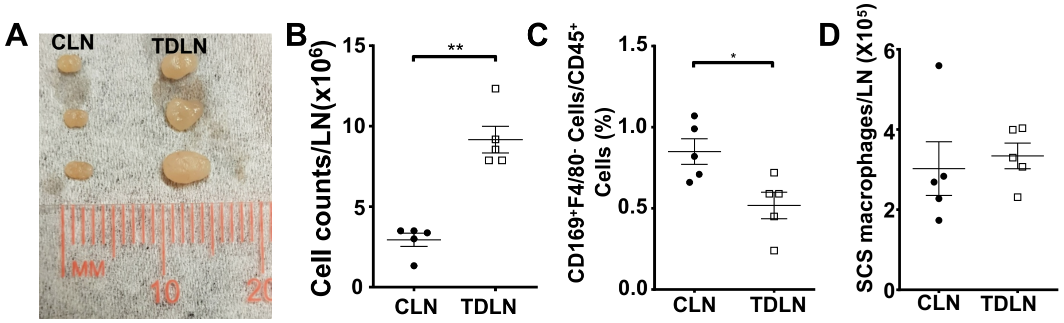

Supplemental Figure 4. A, B. TDLNs were larger than the contralateral LNs. **C,D.** The proportion of SCS macrophages was significantly reduced, but the number of SCS macrophage per LN were comparable between CLN and TDLN 21 post tumor implantation. (n=5 per group, in two separated studies).

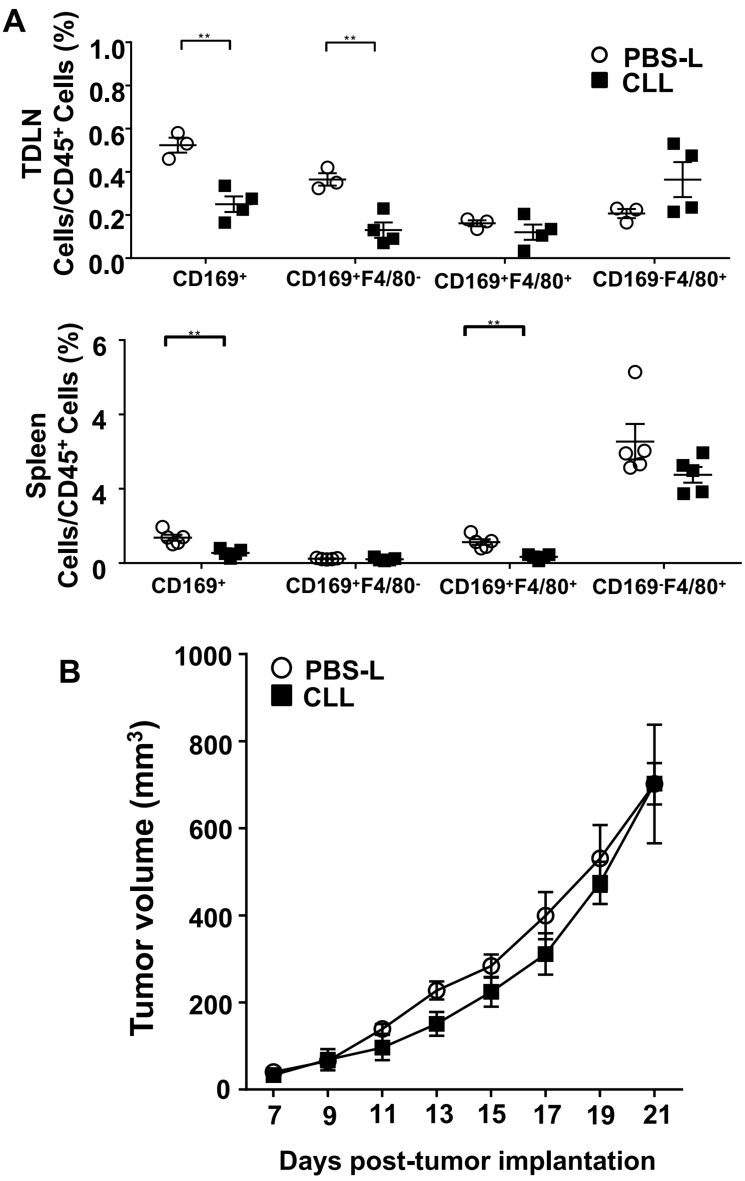

Supplemental Figure 5. Intraperitoneal clodronate liposome treatment depletes macrophages in the spleen and the lymph node but did not affect E0771 tumor growth. A. Flow cytometry analysis of the PBS liposome and clodronate liposome-treated TDLN and the spleen 21 days post-tumor implantation (n=3-5 per group, in two separated studies). Data is mean ± SEM. **p < 0.01. Unpaired, parametric student's t-test. **B.** E0771 growth curve over 21 days of C57BL/6 wild-type mice treated with intraperitoneal systemic injection of 200μL PBS liposomes and clodronate liposomes (n=10 per group, in two separated studies). Data is mean ± SEM. Two-way ANOVA with Sidak's multiple comparisons test.

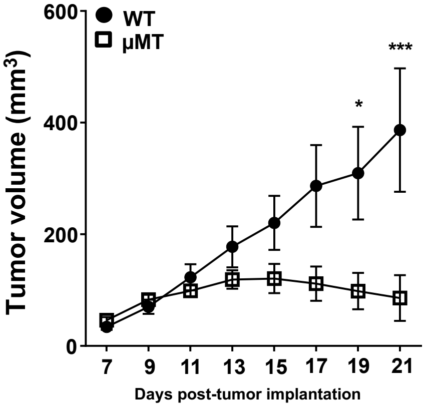

Supplemental Figure 6. E0771-GFP tumor growth was suppressed in B cell deficient (μMT) mice. (n=10 per group, in three separated studies). Data is mean ± SEM. *p < 0.05, ***p < 0.001. Two-way ANOVA with Sidak's multiple comparisons test.
